# Yarn Color Measurement Method Based on Digital Photography

**DOI:** 10.3390/jimaging11080248

**Published:** 2025-07-22

**Authors:** Jinxing Liang, Guanghao Wu, Ke Yang, Jiangxiaotian Ma, Jihao Wang, Hang Luo, Xinrong Hu, Yong Liu

**Affiliations:** 1 School of Computer Science and Artificial Intelligence, Wuhan Textile University, Wuhan 430200, China; jxliang@wtu.edu.cn (J.L.); 2315363151@wtu.edu.cn (G.W.); 2304240309@wtu.edu.cn (K.Y.); 2204241124@wtu.edu.cn (J.M.); 2104240806@wtu.edu.cn (J.W.); hluo@wtu.edu.cn (H.L.); hxr@wtu.edu.cn (X.H.); 2 Key Laboratory of Intelligent Perception and Computing in the Textile Industry, Wuhan 430200, China; 3 College of Art and Design, Wuhan Textile University, Wuhan 430200, China

**Keywords:** yarn color measurement, spectrophotometry, photographic colorimetry, K-means clustering, skeleton extraction, texture weighting, psychophysical experiment

## Abstract

To overcome the complexity of yarn color measurement using spectrophotometry with yarn winding techniques and to enhance consistency with human visual perception, a yarn color measurement method based on digital photography is proposed. This study employs a photographic colorimetry system to capture digital images of single yarns. The yarn and background are segmented using the K-means clustering algorithm, and the centerline of the yarn is extracted using a skeletonization algorithm. Spectral reconstruction and colorimetric principles are then applied to calculate the color values of pixels along the centerline. Considering the nonlinear characteristics of human brightness perception, the final yarn color is obtained through a nonlinear texture-adaptive weighted computation. The method is validated through psychophysical experiments using six yarns of different colors and compared with spectrophotometry and five other photographic measurement methods. Results indicate that among the seven yarn color measurement methods, including spectrophotometry, the proposed method—based on centerline extraction and nonlinear texture-adaptive weighting—yields results that more closely align with actual visual perception. Furthermore, among the six photographic measurement methods, the proposed method produces most similar to those obtained using spectrophotometry. This study demonstrates the inconsistency between spectrophotometric measurements and human visual perception of yarn color and provides methodological support for developing visually consistent color measurement methods for textured textiles.

## 1. Introduction

In the textile production process, accurate color matching between incoming samples and final products critically depends on the precision of yarn color measurement. Human color perception of yarn is influenced by several factors. Individual physiological differences mean color perception varies due to cone cell sensitivity [1]. Also, environmental factors like lighting and viewing angles alter how yarn colors appear [2]. Moreover, yarn’s physical properties, such as uneven dyeing and fiber blends, add to the complexity of color differentiation. Thus, human visual perception of yarn colors is inconsistent and limited. Currently, the industry-standard method primarily relies on spectrophotometers [3], which compute chromaticity values by measuring spectral reflectance within a defined aperture. However, due to the small size of individual yarns, they cannot fully cover the spectrophotometer’s aperture. Consequently, manual yarn winding techniques are commonly employed to expand the measurement area [4,5,6]. This approach, however, presents three technical limitations. First, the measurement results are highly sensitive to process parameters such as the number of winding layers and density—excessive layering tends to overestimate brightness and saturation, while insufficient layering can lead to background interference and reduced accuracy. Second, the directional nature of fiber arrangement and surface texture on yarns results in anisotropic reflection and scattering behaviors, which cannot be effectively captured by the optical system of spectrophotometers, as it is designed based on isotropic assumptions. Third, human visual perception of brightness and color is inherently nonlinear, whereas instrument-based measurements rely on linear models. This fundamental discrepancy hinders accurate representation of human visual experiences.

Vision-consistent imaging-based methods offer a potential solution to the aforementioned limitations. These methods mainly include photographic colorimetry and multispectral imaging. Photographic colorimetry achieves micron-scale chromaticity measurement through high-resolution image acquisition combined with color space transformation algorithms, such as the TDColor system [7], region extraction methods, normal distribution approaches, and kernel density estimation [8], with a reported standard deviation of 1.88 in CIEDE2000 color difference. Multispectral imaging breaks through the RGB channel limitations using spectral decoupling techniques, including Tang’s color matching approach [9], Luo’s average pixel method [10], and Wang’s polynomial model [11]. Notably, Zhang et al. [12] developed the R-Model calibration system using Fréchet distance spectral matching, achieving an average improvement of 54.99% in CIEDE2000 color difference. Kong et al. [13] introduced a feedback-based nonlinear training sample selection strategy, resulting in reconstructed spectral reflectance more closely aligned with actual measurements. Luo et al. [14] proposed four color measurement methods using an MSI system, with the central region averaging method exhibiting the highest brightness stability.

Li et al. [15] advanced beyond traditional unidimensional chromatic analysis by constructing a Spectral Pan-Similarity Metric (SPSM) model to map color features across the full process from dyed polyester fiber to yarn and woven fabric. This model innovatively introduced a chromaticity index weighting algorithm to analyze spectral difference mechanisms between melange yarns and satin fabrics, revealing nonlinear correlations between textile structural parameters and optical responses. Lu et al. [16] developed a bivariate coupling model of pigment mass fraction and fiber linear density to elucidate chromatic evolution from solution-dyed polyester fibers to yarn, providing quantitative benchmarks for process optimization.

Despite improvements in measurement accuracy and stability, two major issues remain. First, the combined effects of multiple light scattering from microstructures on yarn surfaces, complex texture backgrounds [17], and yarn edge effects result in significant brightness deviations, with no effective compensation model currently available. Second, existing chromaticity measurement methods lack a precise mapping to human visual perception. The Weber–Fechner Law [18] and Stevens’ Law [19] collectively demonstrate that human brightness perception follows a piecewise nonlinear pattern—complying with the DeVries–Rose Law in low-brightness regions, a logarithmic relationship in medium-brightness regions, and a power function saturation in high-brightness regions. This nonlinear characteristic leads to difficulty in capturing human sensitivity to low-brightness and saturation at high-brightness levels [20], resulting in considerable discrepancies between measurement results and actual visual perception.

To address these technical bottlenecks in yarn color measurement, this study proposes a centerline-based nonlinear texture-weighted color measurement method, implemented using a self-developed photographic colorimetry system. The method aims to construct a measurement model that aligns with human visual perception. It employs K-means clustering to segment yarn regions and a skeletonization algorithm to extract the yarn centerline, thereby reducing interference from edge texture variations. Spectral reconstruction is then performed along the centerline, and standard chromaticity values are established based on colorimetric theory. Finally, a nonlinear texture-weighted correction based on brightness features is introduced, enhancing the alignment of measurement outcomes with actual visual appearances of yarns. Psychophysical experiments confirm that this method significantly reduces the discrepancy between subjective color perception and objective measurement, greatly improving measurement accuracy and offering a novel approach and technical foundation for the advancement of yarn color measurement technologies.

## 2. Methods

### 2.1. Spectrophotometry (M0)

Spectrophotometry is based on a quantitative conversion model between spectral reflectance and colorimetry. It decomposes incident light into its spectral components using a spectrophotometer, and employs an array-based photodetector to synchronously collect full-spectrum reflectance data. The chromaticity parameters are then calculated via an embedded system [21]. As a foundational experimental step, M0 aims to obtain spectral reflectance data and corresponding chromaticity values of yarns with different colors under varying winding layer conditions, providing benchmark data for subsequent research. In the experiment, yarns with different layer numbers (ranging from one to six layers) are wound onto a standard white cardboard according to a specified method to minimize the impact of background spectra on the measurement results. During the winding process, strict control is maintained over yarn tension and winding uniformity to ensure the surface of the sample is flat and free from overlaps. Additionally, the wound area is required to be no smaller than 5 cm × 5 cm to meet the maximum measurement aperture requirement of the spectrophotometer. Color measurement is carried out using the X-rite Color i7 spectrophotometer, in combination with a computer-based color matching and measurement system for data acquisition. The measurement aperture is set to 25 mm, with a D65 standard light source and a 10° field of view, to conform to standard colorimetric measurement conditions.

### 2.2. Photographic Colorimetry

**Figure 1:**
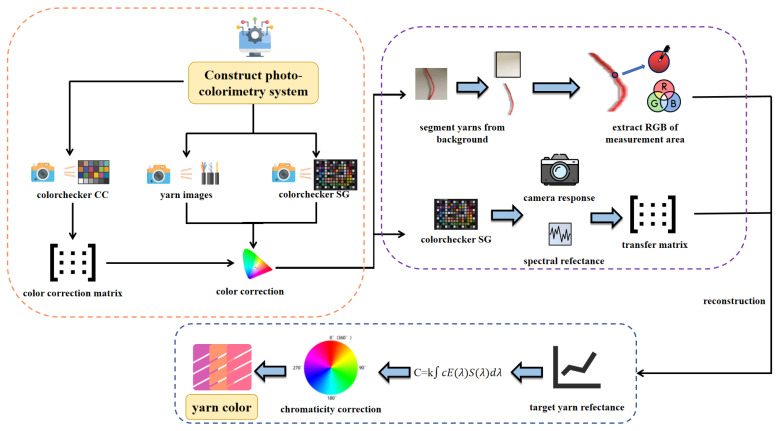
Photographic colorimetry workflow.

Photographic colorimetry initiates with the acquisition of yarn images, then proceeds with color calibration, followed by image segmentation, region extraction, spectral reconstruction, chromaticity calculation, and correction methods, as illustrated in Figure 1, with the pseudocode provided in Algorithm 1. This process ensures that the final measurement results meet high-precision standards in terms of color difference and human visual perception. Additionally, this paper compares and validates the methods of average pixel approach (M1), centerline average pixel method (M2), luminance weighting method (M3), centerline luminance weighting method (M4), texture weighting method (M5), and centerline texture weighting method (M6). The comparison fully demonstrates the superiority and broad applicability of the proposed method in practical applications.
**Algorithm 1:** Photographic Colorimetry Algorithm
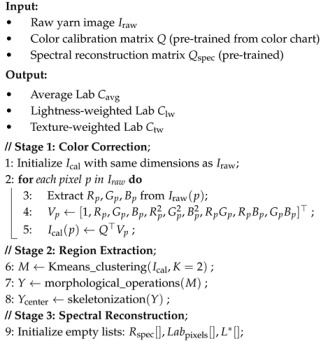

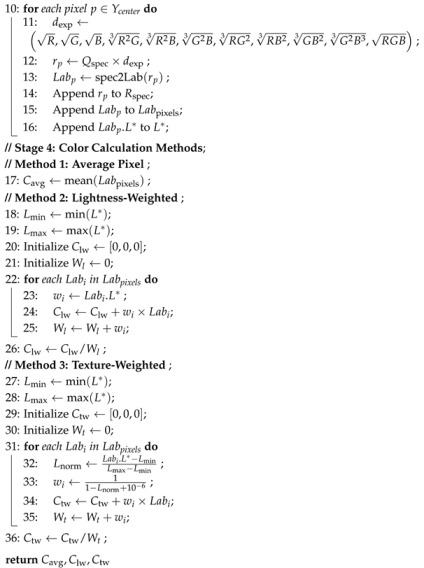


#### 2.2.1. Photographic Colorimetry System

**Figure 2:**
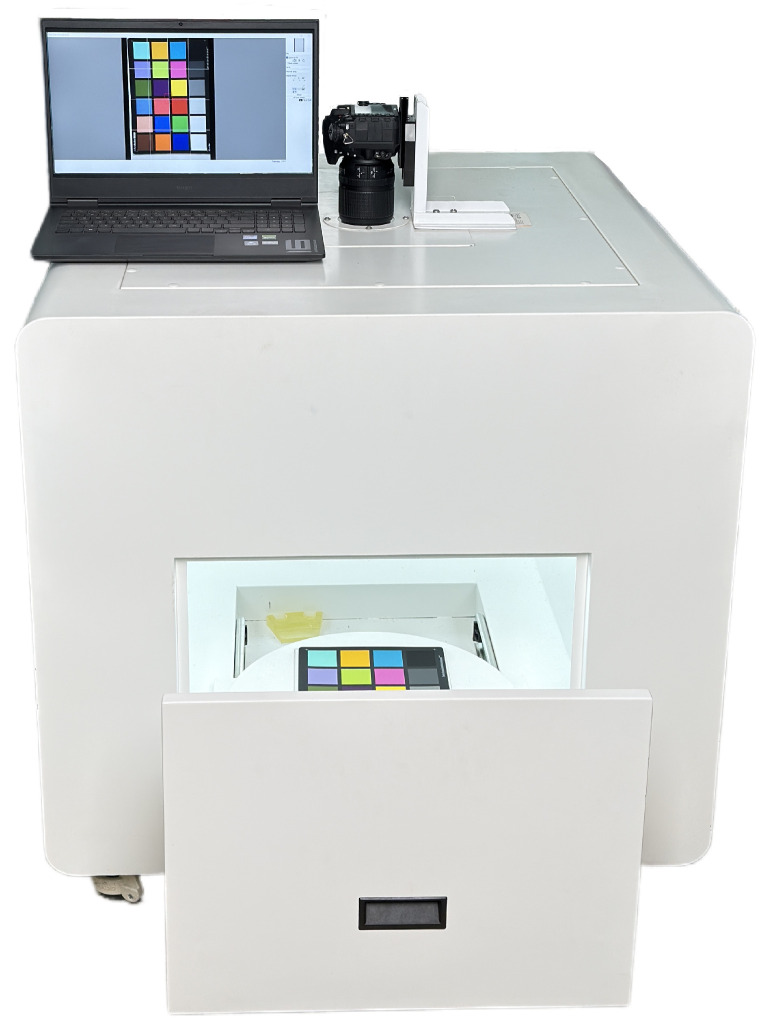
Actual photo of the light box.

The experiment utilizes a self-developed photographic colorimetry system, which includes a closed lightbox (with an integrated D65 standard light source), a Nikon D7200 digital camera (made in Bangkok, Thailand), and an HP Omen 9 computer (made in Chongqing, China). The lightbox provides stable, uniform diffuse illumination, reducing environmental light interference and shadow effects, thereby enhancing image acquisition accuracy and repeatability. The D65 light source is evenly distributed across the top and side walls of the lightbox to achieve omnidirectional uniform illumination, avoiding color measurement deviations caused by uneven illuminance. A physical image of the lightbox is shown in Figure 2. To verify the uniformity of illumination, the X-rite gray card was used to measure the illuminance distribution in the imaging area. The results indicate that the illuminance distribution within the lightbox remains consistent. Camera parameters were calibrated using the X-rite ColorChecker 24 color chart to ensure that the three channels (R, G, B) of the white color block were close to 245, while the three channels of the black color block were close to 50. The final settings were ISO 100 [22], shutter speed 1/25s, aperture f/5.6, and focal length 35mm.

#### 2.2.2. Image Acquisition and Calibration

During the image acquisition process, yarn samples are placed at the center of the sample platform in the lightbox and fixed at both ends using magnets to ensure the yarn remains flat and wrinkle-free. During the shooting process, consistent imaging parameters are used for all samples. Due to the nonlinear spectral response characteristics of digital cameras, directly extracting chromaticity values from the image does not align with standard chromaticity values. To improve color measurement accuracy, the experiment adopts a color calibration method based on a polynomial model [23,24], with the specific implementation method outlined as follows.

(1) By capturing an image of the X-rite ColorChecker 140 color chart, the RGB values of each color block are extracted. A polynomial model is then used to extend the RGB data matrix. Taking the second-order polynomial model as an example, its extended form is shown in Equation (1).

(1) $$V={\left(1,R,G,B,R^{2},G^{2},B^{2},RG,RB,GB\right)}^{T}$$

where *R*, *G*, and *B* correspond to the values of the three color channel components of the color blocks, while *V* represents the color value vector after polynomial expansion.

(2) Based on the standard RGB color data *S* from the color chart and the extended matrix *V* obtained from the captured RGB data of the color chart, the following mapping relationship is established, as shown in Equation (2).

(2) $$S=V\times Q^{T}$$

where *Q* represents the 3 × K-dimensional color correction coefficient matrix to be determined. This matrix is obtained using the least squares method, as shown in Equation (3).

(3) $$Q^{T}=\arg\min_{J}{∥S-QV∥}_{F}^{2}={\left({\left(V^{T}V\right)}^{-1}V^{T}S\right)}^{T}$$

where *J* represents the number of color blocks on the color chart, and ${∥\;\cdot \;∥}_{F}$ denotes the F-norm.

(3) The color correction model *Q* is obtained through Equation (3). The final correction method is shown in Equation (4).

(4) $$I_{c}=IQ^{T}$$

where *I* represents the color data of the captured yarn digital image, *Q* is the color correction model, and $I_{c}$ is the color data of the corrected digital image.

#### 2.2.3. Yarn Photographic Measurement Mode

**Figure 3:**
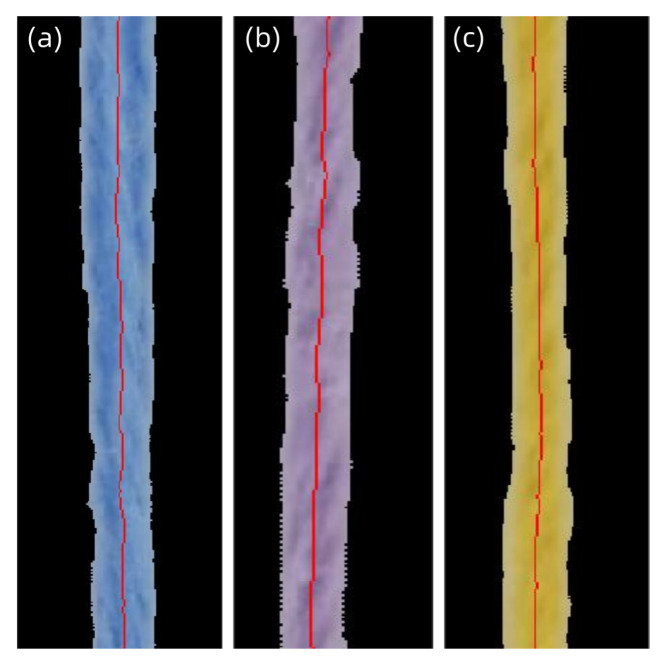
Demonstration of global and centerline extraction effects for yarns of three distinct colors: (**a**) yellow, (**b**) blue, and (**c**) purple.

The yarn color measurement method proposed in this study adopts a dual-path region extraction strategy. First, the K-means clustering algorithm [25] based on the HSV color space is used to segment the overall yarn region. Morphological closing operations are applied to fill holes, and opening operations are used to eliminate discrete noise, thereby constructing a global measurement region that covers the main body of the yarn. On this basis, a multi-stage skeletonization algorithm [26] is further applied to the segmented binary image. Through iterative erosion and topological optimization, a yarn centerline region with a single-pixel width is extracted (as shown in Figure 3), thereby avoiding luminance deviations caused by edge fuzz and surface texture. The two region extraction methods are combined with three chromaticity correction models, forming six measurement modes (as shown in Table 1): the global region and centerline region are respectively paired with the average pixel method (M1/M2), luminance weighting method (M3/M4), and texture weighting method (M5/M6).

##### Average Pixel Method

It is assumed that the pixels in the measurement region contribute equally to the color of the single yarn. The CIELab values of all pixels in the yarn measurement region are directly averaged arithmetically, as shown in Equation (5).

(5) $$C_{\mathrm{avg}}=\frac{1}{N}\sum_{i=1}^{N}C_{i}$$

where *N* is the total number of pixels in the yarn measurement region, *i* represents the i-th pixel in the yarn measurement region, $C_{i}$ is the CIELab value of the i-th pixel, and $C_{avg}$ is the average CIELab value of all pixels in the measurement region.

##### Luminance Weighting Method

By using the luminance of each pixel as the weight, the color of the single yarn can be defined as follows (6).

(6) $$C=\frac{\sum_{i=1}^{N}L_{i}c_{i}}{\sum_{i=1}^{N}L_{i}}$$

where *i* represents the i-th pixel in the measurement region, *N* is the total number of pixels in the measurement region, $L_{i}$ is the luminance value of the i-th pixel, $C_{i}$ is the CIELab value of the i-th pixel, and *C* is the CIELab value after luminance weighting.

##### Texture Weighting Method

M1 assumes that the yarn color is uniformly distributed and does not account for the impact of yarn texture features. Although M3 introduces luminance weighting, the linear mapping model used in it exhibits systematic deviations from the S-shaped response curve of the human visual system (HVS). To address the mismatch between traditional linear weighting methods and human nonlinear visual perception, and based on the Weber-Fechner law and Stevens’ law, it is known that human sensitivity to low-luminance areas is much higher than to high-luminance areas, presenting a clear nonlinear response characteristic. Additionally, due to the anisotropy of fiber arrangement on the yarn surface [27], local highlights may cause luminance distortion. Therefore, this study constructs an inverse proportional weighting function, aiming to enhance the weight distribution of low-luminance areas while suppressing the overexposure interference from highlights, thus more accurately simulating the S-shaped luminance perception curve of the HVS. The specific implementation method is as follows.

(1) The luminance value $L_{i}$ obtained through colorimetric theory is extracted and normalized according to the following formula to obtain the standardized luminance value $\mathrm{normalized}\_L_{i}$, as shown in Equation (7).

(7) $$\mathrm{normalized}\_L_{i}=\frac{L_{i}-L_{\min}}{L_{\max}-L_{\min}}$$

where $L_{\min}$ and $L_{\max}$ represent the minimum and maximum luminance values of the centerline pixels, *i* is the *i*-th pixel on the centerline, and $L_{i}$ is the luminance value of the i-th pixel on the centerline.

(2) Based on the normalized luminance values, the centerline region undergoes texture-weighted correction to calculate the weighted luminance value weighted_L, as shown in Equation (8).

(8) $$\mathrm{weighted}\_L=\frac{1}{1-\mathrm{normalized}\_L+\mathrm{eps}}$$

where normalized_L is the normalized luminance value calculated in Equation (7), and eps is a very small value to avoid a zero denominator.

(3) The final chromaticity value of the yarn is calculated by performing a weighted average of the CIELab values of all pixels in the centerline region using the weighted luminance values, as shown in Equation (9).

(9) $$C_{\mathrm{final}}=\frac{\sum_{i=1}^{N}\mathrm{weighted}\_L_{i}\;\cdot \;C_{i}}{\sum_{i=1}^{N}\mathrm{weighted}\_L_{i}}$$

where *i* represents the *i*-th pixel in the measurement region, *N* is the total number of pixels in the measurement region, $C_{i}$ is the CIELab value of the *i*-th pixel, $\mathrm{weighted}\_L_{i}$ is the weighted luminance value of the *i*-th pixel, and $C_{final}$ is the final CIELab value after texture weighting.

#### 2.2.4. Spectral Reconstruction and Chromaticity Calculation

To further improve the accuracy of yarn color measurement, this study employs a spectral reconstruction method to calculate the spectral reflectance of the yarn, based on the selection of the yarn measurement region. Using root polynomial expansion and the least squares method [28], training is performed with the X-rite ColorChecker 140 color chart, and testing is conducted with the X-rite ColorChecker 24 color chart. The specific implementation method is as follows.

(1) Digital images of the training sample set are captured using the lightbox, ensuring that the shooting conditions are identical to those used for yarn sample photography. The RGB values of each color block are then extracted, as shown in Equation (10).

(10) $$d_{i}=\frac{1}{m\times m}\sum_{j=1}^{m\times m}\left(r_{i,j},\;g_{i,j},\;b_{i,j}\right)$$

where *i* represents the *i*-th color block in the training sample set; *j* represents the *j*-th pixel within the extraction region; $r_{i,j}$, $g_{i,j}$, and $b_{i,j}$ are the red, green, and blue RGB values of the *j*-th pixel in the *i*-th pure color sample, respectively; and $d_{i}$ is the RGB value of the *i*-th color block in the sample set, represented as a 1 × 3 row vector.

(2) The spectral data and RGB values of the training samples are used as input data. The root polynomial expansion method is applied to transform the RGB values, resulting in the extended RGB vector $d_{exp}$, as shown in Equation (11).

(11) $$d_{\exp}=\left(r,g,b,\sqrt{rg},\sqrt{rb},\sqrt[{3}]{gb},\sqrt[{3}]{r^{2}g},\sqrt[{3}]{r^{2}b},\sqrt[{3}]{rg^{2}},\sqrt[{3}]{rb^{2}},\sqrt[{3}]{gb^{2}},\sqrt[{3}]{g^{2}b},\sqrt{rgb}\right)$$

where *r*, *g*, and *b* are the RGB values of the R, G, and B channels of the color block, respectively, and $d_{exp}$ is the extended RGB value vector of a color block.

(3) The spectral reconstruction matrix *Q* is calculated using the least squares method, as shown in Equation (12).

(12) $$Q=R{\left(D^{T}D+\lambda I\right)}^{-1}D^{T}$$

where *R* is the spectral data matrix of the training sample set; *D* is the extended RGB value matrix of the training sample set; *Q* is the spectral reconstruction matrix; *T* denotes the transpose symbol; the superscript “^−1^” represents the inverse operation; λ is the regularization constraint coefficient; *I* is the identity matrix; λI is used to counteract the influence of noise in spectral reconstruction and prevent model overfitting, where λ is typically set to 0.001.

(4) The spectral data of the centerline is calculated, as shown in Equation (13).

(13) r=Qd

where *d* is the extended RGB value of the yarn centerline; *Q* is the spectral reconstruction matrix calculated from Equation (12); *r* is the reconstructed spectral data.

(5) Based on colorimetric theory [29], the reconstructed spectral data is used to calculate the tristimulus values and convert them to the CIELab color space.

## 3. Experimental Results and Analysis

### 3.1. Spectrophotometry Results

**Figure 4:**
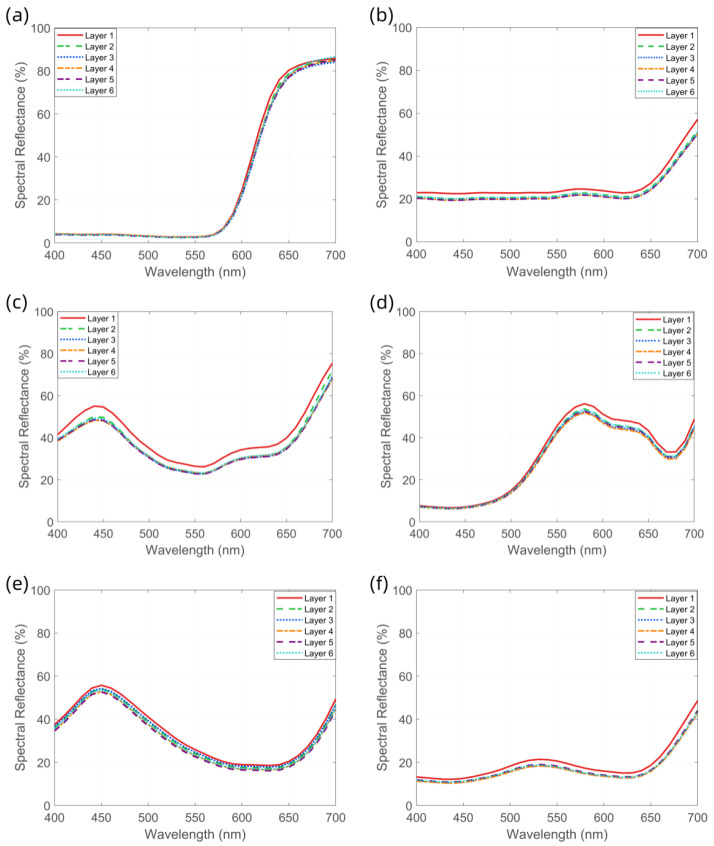
Distribution of spectral measurement results for six different yarn colors under varying winding layers: (**a**) red yarn, (**b**) gray yarn, (**c**) purple yarn, (**d**) yellow yarn, (**e**) blue yarn, and (**f**) green yarn.

**Figure 5:**
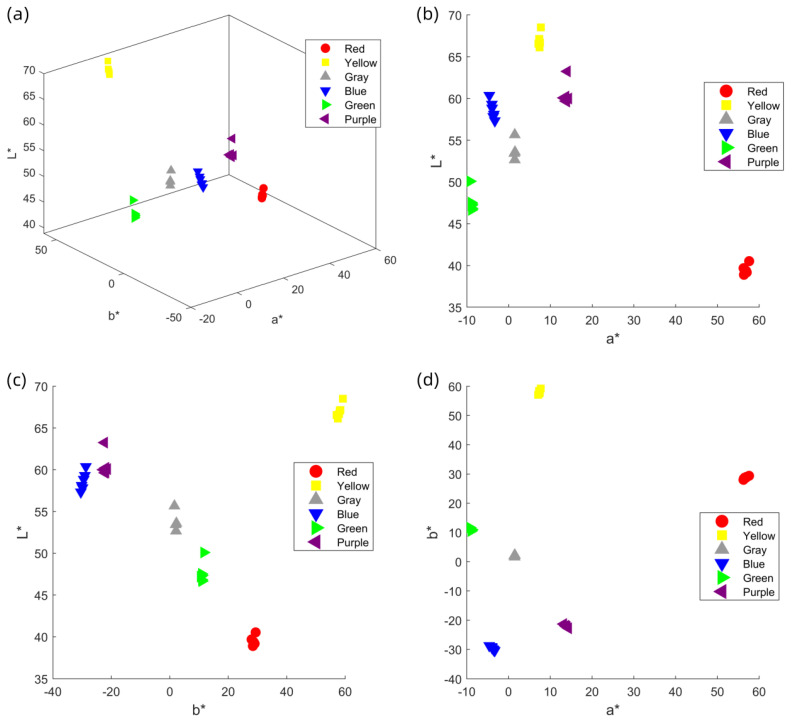
CIELAB chromaticity distribution diagrams of yarn samples with six different colors and varying winding layers: (**a**) L-a-b space, (**b**) L-a plane, (**c**) L-b plane, (**d**) a-b plane.

**Figure 6:**
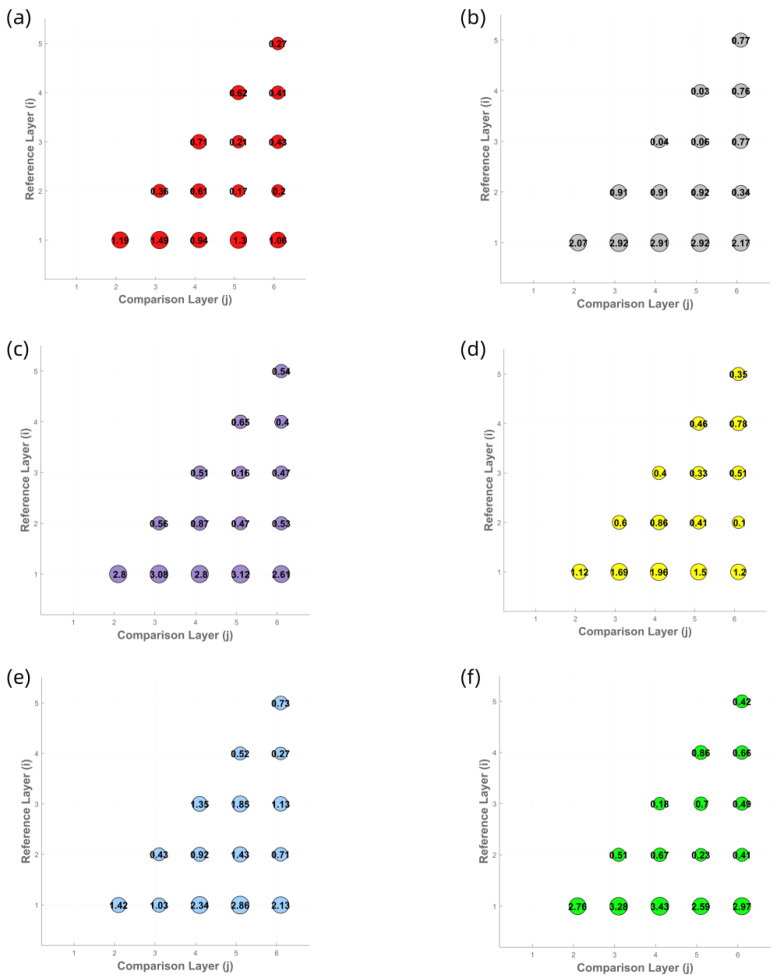
Color difference comparison bubble charts of yarn samples with six different colors and varying winding layers: (**a**) red yarn, (**b**) gray yarn, (**c**) purple yarn, (**d**) yellow yarn, (**e**) blue yarn, and (**f**) green yarn.

To determine the optimal number of yarn winding layers for the M0 method, six different colored yarns (all made of cotton with a count of 32) were used to measure the spectral reflectance and CIELab chromaticity values of samples with one to six layers. Corresponding spectral reflectance curves (Figure 4), CIELab chromaticity distribution maps (Figure 5), and color difference comparison charts (Figure 6) were generated to evaluate the influence of winding layer count on the stability of color measurements.

The experimental results indicate that the spectral reflectance curves become progressively more consistent with the increase in winding layers. For samples with four or more layers, the spectral reflectance curves across all colors converge to a stable state, suggesting that the spectral characteristics of the yarn colors have stabilized. This trend is further confirmed by the CIE chromaticity distribution maps, where the chromaticity coordinate points of samples with four or more layers are closely clustered. Moreover, the $\Delta E_{00}$ values between the four-, five-, and six-layer samples for all six yarn colors are below 1.0, which complies with the commercial-grade acceptability threshold of $\Delta E_{00}\leq 1.0$ in the textile industry. In contrast, samples with fewer than four layers exhibit significantly higher $\Delta E_{00}$ variation, indicating that their color measurements are still influenced by surface texture and background interference. Based on the analysis of the spectral reflectance curves and chromaticity distributions, it is concluded that the four-layer winding samples can accurately reflect both the spectral and visual chromatic characteristics of the yarns. Therefore, four-layer winding samples are selected as the benchmark data for the M_0_ method in subsequent experiments.

### 3.2. Photographic Colorimetry Results

**Figure 7:**
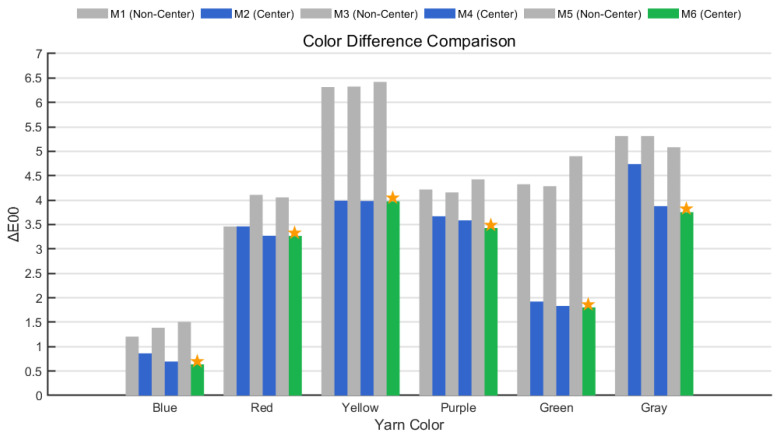
Comparison of average color differences between six different photographic color measurement methods and spectrophotometer measurements. The star sign denotes the photographic method that yields the smallest color difference for each yarn color when compared to the spectrophotometric measurement.

In this experiment, six yarn samples with different colors—corresponding to those used in the M0 method—were evaluated using six photographic colorimetry methods (M1 through M6). The CIELab values obtained from each method were compared with the corresponding M0 results to calculate the CIEDE2000 color differences ($\Delta E_{00}$), thereby providing a preliminary assessment of the deviation between photographic and spectrophotometric color measurement methods. The specific results are presented in the following Table 2, Table 3 and Table 4 and in Figure 7.

Systematic analysis of the experimental data reveals that the skeletonization algorithm (centerline region) effectively suppresses surface texture interference and edge light scattering. Compared with global regions, it significantly reduces the average color differences between the photographic methods and the spectrophotometric reference across all six yarn colors. Under the average pixel model, transitioning from M_1_ to M_2_ results in a 25.36% reduction in overall $\Delta E_{00}$ (from 4.1354 to 3.0864). Notably, the $\Delta E_{00}$ for the green yarn decreases from 4.3249 to 1.9239—a 55.5% reduction.

In the luminance-weighted model, moving from M_3_ to M_4_ yields a 34.21% decrease in average color difference (from 4.26 to 2.87). Specifically, the $\Delta E_{00}$ for the red yarn drops from 4.1082 to 3.271 (a 20.4% reduction), while the blue yarn—representing a cool tone—shows a $\Delta E_{00}$ decline from 1.3878 to 0.6962 (a 49.8% reduction), closely approaching the M_0_ results. The gray yarn also benefits from the centerline algorithm, which eliminates edge shadow interference and enhances luminance values, thus demonstrating the model’s effectiveness in improving chromaticity accuracy for neutral tones.

In the texture-weighted model, a comparison between M_5_ and M_6_ indicates that M_6_ further suppresses overexposure in high-luminance areas via an inverse-proportional weighting function. This adjustment reduces the average color difference across all six yarn colors from 4.3989 (M_5_) to 2.8104 (M_6_), marking a 36.11% reduction. For the blue yarn, the $\Delta E_{00}$ drops from 1.5077 (M_5_) to 0.6373 (M_6_), nearly matching the spectrophotometric result.

From the perspective of model progression, the $\Delta E_{00}$ values demonstrate a stepwise reduction across the M_2_→ M_4_→ M_6_ sequence (with global means decreasing from 2.31 to 2.13), confirming the effectiveness of the nonlinear texture-weighted correction method for addressing the nonlinear response characteristics of yarn surface texture. The $\Delta E_{00}$ for the blue yarn (0.6373) is almost identical to the M_0_ result, while the green yarn’s $\Delta E_{00}$ of 1.803 approximates the reference. Although the $\Delta E_{00}$ for warm-toned yarns remains slightly higher, the M_6_ method still produces results closer to the spectrophotometric measurements compared to the other five methods. This demonstrates that the proposed approach can consistently obtain chromaticity data comparable to spectrophotometric results without requiring the yarn winding process, thereby offering objective support for developing color measurement techniques aligned with human visual perception.

### 3.3. Subjective Evaluation of Measurement Results

**Figure 8:**
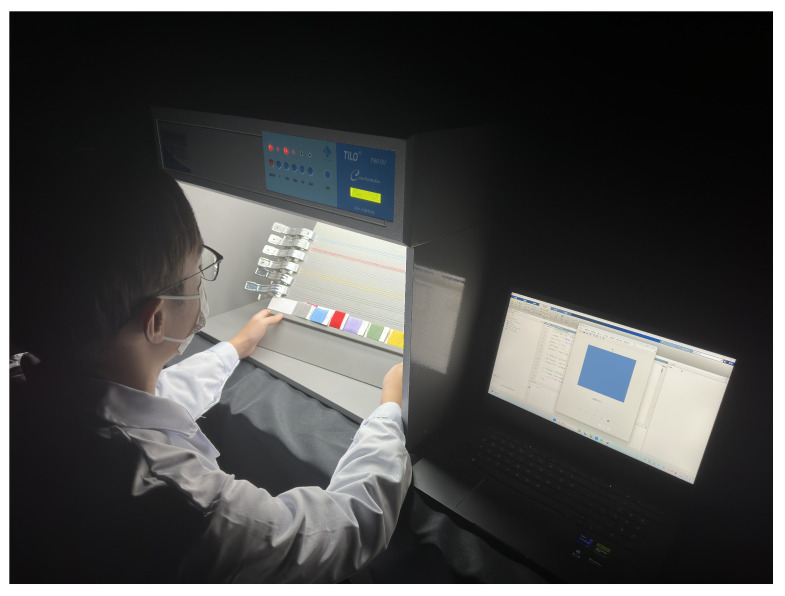
Schematic diagram of a psychophysics experiment scenario.

To validate the visual perception consistency of the yarn color measurement methods, a psychophysical experiment was designed and conducted under strictly controlled environmental conditions using standardized display equipment and a viewing booth. The display presented the yarn colors as measured by different methods, while the viewing booth contained the six yarn samples and their corresponding four-layer winding samples, as illustrated in Figure 8. After calibration, the display device achieved an average CIEDE2000 color difference of 1.0 for 24 standard color patches, ensuring color accuracy during the experiment.

A total of 20 observers, all having passed a color vision screening, participated in the evaluation to ensure normal color discrimination capabilities. Prior to the formal test, each observer underwent an adaptation phase in a darkroom environment to stabilize their visual perception. During the evaluation, observers compared the yarn color displayed on the screen with the actual samples placed in the booth and rated the perceived similarity using a 7-point Likert scale, where 1 indicated the least similarity and 7 indicated the highest. Each observer completed three rounds of scoring to ensure the reliability and consistency of the results.

**Figure 9:**
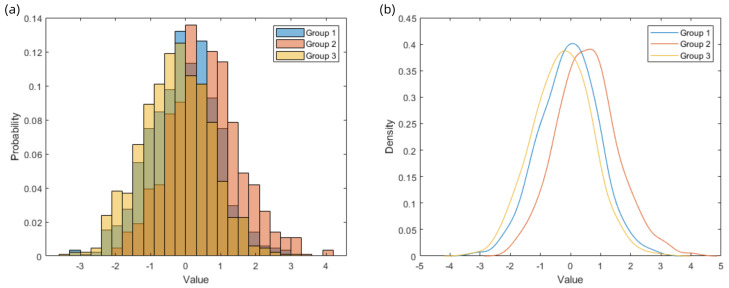
(**a**) Probability histogram: Group 1 and Group 2 exhibit similar variances, while Group 3 shows slightly greater dispersion; (**b**) Kernel density estimation plot: Group 1 has a mean near zero, whereas Groups 2 and 3 exhibit noticeable shifts.

**Figure 10:**
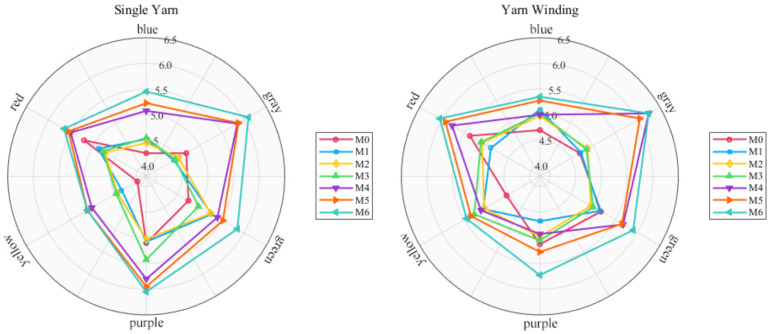
Comparison of visual perception consistency evaluation results for six yarn colors and their wound samples.

Upon completion of the experiment, the data from all three rounds were collected and subjected to analysis of variance (ANOVA). The results revealed a statistically significant difference among the three rounds (*p* = 0.0014), with the third round yielding significantly higher mean scores than the first two. However, no significant variation was found within each round across different observers. After standardization, the variance analysis indicated no longer significant differences between groups, and subsequent evaluations focused on the first two rounds to better reflect the participants’ scoring trends. The average scores of the 20 participants are shown in Table 5, as well as Figure 9 and Figure 10.

Visual perception assessments based on the psychophysical data indicate that for the evaluation of single yarn samples, the M0 method received an average score of 4.667. Although it slightly outperformed the traditional global region-based methods for red (5.2) and gray (4.7) yarns (compared to M1 = 4.754, M3 = 4.738, M5 = 4.775), it was significantly outscored by all centerline-based methods (M2 = 5.442, M4 = 5.542, M6 = 5.704). The M6 method achieved the highest overall average score of 5.704, strongly validating the close alignment between the centerline texture-weighted measurement method and human visual perception.

For the evaluation of winding samples, M0 showed slight advantages in red (5.375) and green (5.175) samples, but its performance was comparable to the traditional global methods (M1 = 4.938, M3 = 4.988, M5 = 5.054). In contrast, all centerline-based methods (M2 = 5.458, M4 = 5.588, M6 = 5.788) exhibited superior perceptual consistency. Notably, M6 again achieved the highest score of 5.77. Its score for the winding samples even surpassed that for single yarn samples, indicating the robustness of this method in handling complex textile structures.

Overall, the subjective evaluation data demonstrate that the linear measurement model of spectrophotometry cannot fully reflect human visual perception. In contrast, photographic colorimetry aligns more closely with the nonlinear characteristics of human color and luminance perception. Specifically, the use of centerline regions effectively reduces surface texture and edge scattering interference, resulting in more concentrated and stable measurement areas. Furthermore, the texture-weighted strategy, by simulating the S-shaped response curve of the human visual system—sensitive to low luminance and saturated in high luminance—achieves a more accurate perceptual correction.

## 4. Conclusions

This study proposes a yarn color measurement method based on centerline texture-weighting and reveals, through psychophysical experiments, the limitations of traditional spectrophotometry in achieving visual perception consistency. Experimental results demonstrate that the visual consistency of spectrophotometry is significantly lower than that of photographic colorimetry. By selecting the yarn centerline as the measurement region, the proposed method effectively reduces interference from surface texture and edge scattering. Combined with a nonlinear texture-weighting model that simulates the S-shaped luminance response of the human visual system, the method achieves an improved alignment between objective measurement accuracy and subjective visual perception. Moreover, the method eliminates the need for yarn winding while producing measurement results that are numerically comparable to those of spectrophotometry but more aligned with actual human perception. Although the M6 method demonstrates the best performance among the evaluated approaches, its results still fall short of fully replicating human visual perception. Future work will focus on developing more advanced algorithms to further narrow the gap between objective measurement and subjective visual experience.

## Figures and Tables

**Table 1:** Combination of measurement area and measurement method model.

	Area	Global	Centerline
Method			
Average Pixel		M1	M2
Luminance Weighting		M3	M4
Texture Weighting		M5	M6

**Table 2:** CIELAB values obtained from global averaging (M1) and centerline averaging (M2) of yarn samples, along with their color differences compared to spectrophotometer measurements (M0).

Yarn Color	M0			M1			$\Delta E_{00}$	M2			$\Delta E_{00}$
	$L^{*}$	$a^{*}$	$b^{*}$	$L^{*}$	$a^{*}$	$b^{*}$		$L^{*}$	$a^{*}$	$b^{*}$	
blue	57.84	−3.68	−29.85	57.88	−3.30	−27.07	1.2062	56.95	−3.10	−29.35	0.8633
red	39.68	56.26	28.00	42.12	48.82	28.03	3.4596	41.00	52.99	32.50	3.3363
yellow	66.12	7.39	57.52	70.19	−0.07	48.40	6.3140	68.18	1.98	58.90	3.9889
purple	60.08	13.16	−21.33	60.99	10.92	−13.83	4.2172	57.84	13.46	−16.38	3.6691
green	46.67	−8.86	11.02	50.10	−8.97	15.18	4.3249	48.62	−8.87	11.08	1.9239
gray	52.67	1.45	2.14	51.28	−0.27	7.35	5.2905	56.01	−0.33	4.70	4.7371

**Table 3:** CIELAB values obtained from global luminance-weighted (M3) and centerline luminance-weighted (M4) measurements of yarn samples, along with their color differences compared to spectrophotometer measurements (M0).

Yarn Color	M0			M3			$\Delta E_{00}$	M4			$\Delta E_{00}$
	$L^{*}$	$a^{*}$	$b^{*}$	$L^{*}$	$a^{*}$	$b^{*}$		$L^{*}$	$a^{*}$	$b^{*}$	
blue	57.84	−3.68	−29.85	58.26	−3.14	−26.69	1.3878	57.15	−3.08	−29.23	0.6962
red	39.68	56.26	28.00	43.07	48.60	27.98	4.1082	41.18	53.42	32.49	3.271
yellow	66.12	7.39	57.52	70.29	0.16	47.65	6.3233	68.24	1.98	58.37	3.9814
purple	60.08	13.16	−21.33	60.96	11.03	−13.92	4.1582	58.07	13.44	−16.32	3.5854
green	46.67	−8.86	11.02	50.10	−8.94	15.08	4.2859	48.53	−8.85	11.06	1.833
gray	52.67	1.45	2.14	51.5	−0.31	7.43	5.3123	55.57	−0.13	0.51	3.8775

**Table 4:** CIELAB values obtained from global texture-weighted (M5) and centerline texture-weighted (M6) measurements of yarn samples, along with their color differences compared to spectrophotometer measurements.

Yarn Color	M0			M5			$\Delta E_{00}$	M6			$\Delta E_{00}$
	$L^{*}$	$a^{*}$	$b^{*}$	$L^{*}$	$a^{*}$	$b^{*}$		$L^{*}$	$a^{*}$	$b^{*}$	
blue	57.84	−3.68	−29.85	58.39	−3.16	−26.53	1.5077	57.22	−3.09	−29.19	0.6373
red	39.68	56.26	28.00	42.99	48.68	28.09	4.0565	41.14	53.43	32.51	3.266
yellow	66.12	7.39	57.52	70.38	0.11	47.38	6.4206	68.31	2.03	58.38	3.9764
purple	60.08	13.16	−21.33	61.39	10.76	−13.61	4.4256	58.15	13.23	−16.40	3.4289
green	46.67	−8.86	11.02	50.87	−8.76	14.92	4.8995	48.5	−8.852	11.04	1.803
gray	52.67	1.45	2.14	52.24	−0.26	7.30	5.0834	55.42	−0.09	0.48	3.7522

**Table 5:** Psychophysical experiment scores of six yarn colors and their winding samples.

Method	Single Yarn			Yarn Winding		
	First	Second	Average	First	Second	Average
M0	4.625	4.708	4.667	4.842	5.033	4.938
M1	4.717	4.792	4.754	4.850	5.025	4.9375
M3	4.750	4.725	4.738	4.975	5.000	4.968
M5	4.742	4.808	4.775	4.992	5.117	5.054
M2	5.450	5.433	5.442	5.417	5.500	5.458
M4	5.417	5.667	5.542	5.617	5.558	5.588
M6	5.617	5.792	5.704	5.808	5.767	5.788

## Data Availability

The data used to support the findings of this study are available from the corresponding author upon request.

## Abbreviations

Mathematical symbols used in yarn color measurement research

**Symbol**	**Type**	**Definition**
R,G,B	Variables	RGB channel values of color blocks
*V*	Vector	Expanded color value vector
*S*	Matrix	Standard RGB data matrix from color chart
*Q*	Matrix	Color correction coefficient matrix
*I*	Matrix	Original image color data
$I_{c}$	Matrix	Corrected image color data ($I_{c}=IQ^{T}$)
${∥\;\cdot \;∥}_{F}$	Operator	Frobenius norm for matrix computation
$L_{i}$	Variable	Luminance value of the i-th pixel
$L_{\min}$	Constant	Minimum luminance value in centerline pixels
$L_{\max}$	Constant	Maximum luminance value in centerline pixels
$\begin{array}{c} \mathrm{normalized}\_L_{i} \end{array}$	Variable	Normalized luminance value: $\frac{L_{i}-L_{\min}}{L_{\max}-L_{\min}}$
$\begin{array}{c} \mathrm{weighted}\_L_{i} \end{array}$	Variable	Texture-weighted luminance: $\frac{1}{1-\begin{array}{c} \mathrm{normalized}\_L_{i} \end{array}+\mathrm{eps}}$
$C_{i}$	Vector	CIELab value of the i-th pixel
$C_{\mathrm{final}}$	Vector	Final CIELab value: $\frac{\sum_{i=1}^{N}\begin{array}{c} \mathrm{weighted}\_L_{i}\;\cdot \; \end{array}C_{i}}{\sum_{i=1}^{N}\begin{array}{c} \mathrm{weighted}\_L_{i} \end{array}}$
eps	Constant	Small epsilon value ($\approx 10^{-6}$) to prevent division by zero
*N*	Constant	Total number of pixels in measurement region
$d_{i}$	Vector	RGB value of i-th color block
$d_{\exp}$	Vector	Expanded RGB value vector
*R*	Matrix	Spectral data matrix
*D*	Matrix	Expanded RGB value matrix
*r*	Vector	Reconstructed spectral data
λ	Constant	Regularization coefficient (λ=0.001)
